# Herd Level Yield Gap Analysis in a Local Scale Dairy Farming System: A Practical Approach to Discriminate between Nutritional and Other Constraining Factors

**DOI:** 10.3390/ani13030523

**Published:** 2023-02-02

**Authors:** Igino Andrighetto, Lorenzo Serva, Davide Fossaluzza, Giorgio Marchesini

**Affiliations:** Dipartimento di Medicina Animale, Produzioni e Salute, Università degli Studi di Padova, 35020 Legnaro, Italy

**Keywords:** dairy cow, production ecology, income over feed cost, digestible dry matter, limiting factors

## Abstract

**Simple Summary:**

Improving feed efficiency is one of the keys to meet the production and economic needs of dairy farmers and reduce environmental impact; however, reaching this goal is a challenge. Our results showed that farms with low and average production and efficiency must improve their feed quality to increase the amount of feed digested by cows. In addition, low producing farms must eliminate the factors which reduce the conversion of digested feed into milk, such as negative health events, distress and management procedures. This study provides some indicators and a practical approach to help farmers understand their main limitations to reaching achievable milk yield.

**Abstract:**

This study performed a yield gap analysis to help farmers understand whether their constraints were mainly due to nutritional factors or management and health issues. Twenty-nine farms were periodically evaluated. Milk yield (MY), dry matter intake (DMI), total mixed ration (TMR) composition and homogeneity index (HI), TMR digestibility, income over feed cost (IOFC), and MY summer–winter ratio (SWR) were collected. Farms were divided and compared according to the average annual MY: Low (L), Medium (M) and High (H), characterised by <31.1, 31.1–36.7 and >36.7 kg/head/day. An ANOVA mixed model and a stepwise regression to assess the relationship between nutritional variables and MY were run. H farms showed higher IOFC (*p* < 0.001), DMI (*p* = 0.006), DDM (*p* < 0.001), digestible crude protein (DCP, *p* = 0.019), HI (*p* = 0.09), SWR (*p* = 0.041) and lower HI coefficient of variation (*p* = 0.04). The conversion of DDM into milk was higher in H and M farms. Stepwise regression for MY selected DDM and CP (R^2^ = 0.716, *p* < 0.05). M farms were mainly constrained by nutritional factors, whereas L farms were also affected by other factors such as those related to management and health.

## 1. Introduction

In dairy farming, efforts are being made to increasingly combine the production and economic needs of farmers with those of reducing environmental impact and preserving animal welfare and health [1]. Indeed, it is expected that the world demand for milk and more generally for products of animal origin will increase by at least 70% over the next 30 years [2]. This estimate consequently raises the question on how to meet this demand and achieve high-quality production that is affordable for farmers, is sustainable, and is respectful of animal health and welfare. One of the keys to reach this goal is to improve feed efficiency (FE), defined as the ratio between milk yield and feed intake [3]. FE is in fact considered an important factor affecting the overall efficiency in milk production [3]. The improvement of FE should be one of the main goals of dairy farms to improve profitability [3], since feed represents the highest cost input. Income Over Feed Cost (IOFC), which is the outcome of the difference between milk income and the feed costs, represents a good metric for the assessment of profit margin, since it considers the volatility of both milk and feed prices [4,5]. However, improving FE and thus profitability for a farmer is a very challenging task, since there is no constant conversion efficiency of dry matter intake (DMI) into milk yield and understanding which of the many factors involved are the most limiting is not easy.

In this regard, a detailed yield gap analysis could help farmers to estimate the extent to which their production, FE and IOFC can be increased [6]. Yield gap analyses are used to estimate the difference between the potential (maximum achievable) and actual production of an individual animal, a farm or a region and describe the possible causes of this difference [6]. In a practical context, potential production can be replaced by the attainable production, where attainable production is the maximum yield achievable given locally available technologies and resources [7]. According to production ecology, livestock production is determined by defining, limiting, and reducing factors [7]. Genotype and climate represent the defining factors which determine the potential production; quality and quantity of feed and water are the limiting factors, which lead to the so-called feed-limited production; health issues, distress and management procedures are considered the reducing factors [7], which are further factors responsible for the decrease of milk production from the value estimated for set nutritional factors (feed-limited) to the actual level [7].

With regard to the limiting factors, forage quality, diet fibre concentration and energy level mainly affect DMI, which is a well-known driver of milk yield. Some factors change the metabolic partitioning of energy and protein through the shift of insulin sensitivity and hormonal profile [8]; others affect digestibility, through the modification of rumen motility and microbiome [3]. For example, total mixed ration (TMR) chemical and physical homogeneity [9,10], forage particles length and adhesiveness between long and fine TMR particles are reported to affect animals’ sorting activity and change the effectiveness of feed utilization by rumen microbial consortia [9,11]. A factor that effectively summarizes the effect of all the nutritional variables and is very useful for monitoring FE is total tract diet digestibility, here referred to as digestibility, which includes both the effects of the intrinsic feed digestibility and the cow’s digestive function [12].

Regarding reducing factors, at the herd level, attention should be paid to all those physiological parameters related to milk production, such as the herd’s average days in milk, parity or percentage of primiparous cows [3,13]. In addition, all the facilities’ characteristics and management options which can affect the animals’ health status or cause distress must be taken into consideration; some examples are the number and quality of cubicles, space available at the feed bunk, time budget, indoor environment and social conditions [14,15]. There are a number of methods reported in the literature for calculating potential or attainable yields and conducting a yield gap analysis [6]. Van Der Linden et al. [7], for instance, developed a mechanistic model combining the effects of defining (genotype, climate) and limiting (feed quality and quantity) factors to calculate potential and feed-limited milk yield in individual dairy cows, but they did not address reducing factors. This study aimed to identify some indicators that, on a local scale, considering similar farms for breed, environmental conditions and feeding system, could help farmers calculate their yield gap and understand whether their constraints are mainly due to limiting (nutritional) or reducing factors.

## 2. Materials and Methods

### 2.1. Ethical Statement

The trial was carried out in commercial farms without using or manipulating any animals. Data related to animal performance were gathered by farmers’ management software. Faeces collected to calculate total tract digestibility were gathered from the ground upon their production, without manipulating the animals.

### 2.2. Experimental Design and Farms’ Characteristics

The trial was carried out on commercial farms without using or manipulating animals. The research was an observational open cohort study involving 29 dairy cow farms located in the Po Valley between the regions of Lombardy (provinces of Brescia, Cremona and Mantova) and Veneto (provinces of Vicenza, Padova and Verona), the first and third Italian regions for milk production, respectively [16]. Farms, all raising Italian Holstein cows with the characteristics reported in Table 1, were recruited on a voluntary basis from a list of eligible farms raising Holstein cows provided by feed companies operating in the research area.

On all farms, animals were raised in loose housing facilities, with cubicles in the resting area. All the farms housed milking cows in a single group, which is a common practice on commercial dairy farms, as also reported by Contreras-Govea et al. [17], and fed them a TMR based on maize silage and alfalfa hay or silage. Among the other components of the ration, farmers used winter cereal silages (ryegrass, wheat, barley), grass hays, straw, maize (meal or high-moisture maize grain silage), soybean or sunflower extracted meal and mixed minerals and vitamins. Farms were visited by research personnel an average of 4.89 times each, on a seasonal basis, mainly in summer and winter, over a two-year period (2019–2020). Trained researchers, supported by the farm personnel, collected each farm’s basic data (number of cows in milk, herd average days in milk, ration fed to milking cows), TMR and faecal samples and performed on-farm analyses on TMR characteristics (Table 2) through a PoliSPEC^NIR^ (ITPhotonics, Fara Vicentina, Italy) spectrophotometer.

### 2.3. Herd Production, Digestibility and Feed Monitoring

At each farm visit, as described below, data on the average daily individual milk yield and average feed intake were recorded, and samples of TMR and faeces were collected to determine chemical and physical TMR composition and total tract digestibility [18]. Furthermore, TMR was analysed through a portable near infrared spectrophotometer (PoliSPEC^NIR^, ITPhotonics, Fara Vicentina, Italy) and a robust calibration curve (Table 2) to measure the TMR homogeneity index (HI) as reported in the literature [10].

The average individual daily milk yield was based on the daily production in the three running days preceding the visit. Feed intake for the lactating cow group was calculated as the difference between TMR distributed in the morning of the visit and the leftovers weighed after 24 h, divided by the number of cows; the outcome was corrected by the DM of the TMR collected at the day of the visit, to estimate the dry matter intake (DMI). Total tract digestibility of DM (DMD), CP, NDF and starch was determined using acid detergent lignin as an internal marker [18], according to the following formulas:

(1) $$\mathrm{DMD}=\frac{\mathrm{marker}\;\mathrm{in}\;\mathrm{faeces}\;\left(g/\mathrm{kg}\;\mathrm{DM}\right)-\;\mathrm{marker}\;\mathrm{in}\;\mathrm{feed}\;\left(g/\mathrm{kg}\;\mathrm{DM}\right)}{\mathrm{marker}\;\mathrm{in}\;\mathrm{faeces}\;\left(g/\mathrm{kg}\;\mathrm{DM}\right)}$$

(2) $$\mathrm{ND}=1-\frac{\mathrm{marker}\;\mathrm{in}\;\mathrm{feed}\;\left(g/\mathrm{kg}\;\mathrm{DM}\right)\times \;N\;\mathrm{in}\;\mathrm{faeces}\;\left(g/\mathrm{kg}\;\mathrm{DM}\right)}{\mathrm{marker}\;\mathrm{in}\;\mathrm{faeces}\;\left(g/\mathrm{kg}\;\mathrm{DM}\right)\times \;N\;\mathrm{in}\;\mathrm{feed}\;\left(g/\mathrm{kg}\;\mathrm{DM}\right)}$$

where N is the nutrient and ND is the nutrient digestibility.

Samples of TMR and faeces were immediately refrigerated after collection and analysed upon arrival to the laboratory through the use of a NIR FOSS DS-2500 scanning monochromator (FossNIR-System, Silver Spring, MD, USA) and robust calibration curves reported in Table 2. The TMR sample (~3 kg) was collected upon its distribution to the animals, merging multiple subsamples along the feeding line. Fresh faecal samples were immediately collected from the ground after deposition, 4 h after the morning distribution of TMR, from 12 cows between 50 and 120 days in milk; 12 was considered the minimum reliable sample size needed to find differences in total tract digestibility, obtained through statistical power analysis, as reported by Tharangani et al. [19]. Faecal sampling time was based on the fact that there is slight variation in concentration of ADL in daily faecal excretion [20] and daily average indigested fibre content is particularly similar to that measured 4 h after the distribution of the meal [8].

The measure of TMR homogeneity was carried out through a patented procedure [10] and through the use of a portable NIR instrument (PoliSPEC^NIR^) pre-calibrated (Table 2) for CP, NDF, starch, geometric mean particle length (GMPL) and the percentages of TMR present in the last three layers of a 6-level sieve. The sieve used to build the calibration curve was a modification of the Penn State Particle Separator with, in addition to the bottom pan, 5 sieves with holes with a diameter of 38.1 mm (sieve 1), 19.1 mm (sieve 2), 7.9 mm (sieve 3), 3.8 mm (sieve 4) and 1.8 mm (sieve 5), respectively [10]. Briefly, NIR scans were carried out immediately after TMR distribution along the whole feeding line at regular intervals in 16 representative spots (4 spots per each of the 4 sectors in which the feeding line was imaginarily divided). The relevance of the variations between sectors of the individual variables (CP, NDF, starch, GMPL, sieves 4–5 and bottom pan) determines the attribution of a weighed score to each variable through the application of an algorithm which, as final outcomes, calculates the homogeneity index (HI) ranging from 0 to 100, where 0 corresponds to a totally inhomogeneous TMR and 100 to a totally homogeneous TMR [10].

### 2.4. Sample Analysis to Build Calibration Curves

The particle size distribution of TMR samples was obtained by sieving the samples (two replicates) through the modified Penn State Particle Separator described above, which was filled with wet TMR and shaken as reported by Heinrichs [21]. The GMPL was calculated as reported by the American Society of Agricultural and Biological Engineers, ASABE [22]. The TMR nutrient composition to build the NIR calibration curves was determined through the following procedures: 934.01 for DM, 2001.11 for CP, #996.11 for starch [23] and ANKOM Technology [24] for NDF (with amylase and sodium sulfite), ADF and ADL.

### 2.5. Calculations and Statistical Analysis

The statistical analyses, with the exception of summer–winter ratio (SWR), were performed after averaging all data within farms to obtain more robust data and avoid the effect of the daily visit. Farms, on the basis of their average daily milk yield per cow, given the wide range between the most and least productive farms, were divided into three production classes: Low production (group L, N = 5; 17.2% of total farms), with milk production <31.1 kg/head/day; Medium production (group M, N = 19; 65.6% of total farms) with production between 31.1 and 36.7 kg/head/day; and High production (group H, N = 5; 17.2% of total farms) with production > 36.7 kg/head/day. The milk production thresholds chosen to define the L, M and H groups were calculated as overall average milk production minus standard deviation (mean − SD = 31.1 kg/head/day) and overall average milk production plus standard deviation (mean + SD = 36.7 kg/head/day), respectively.

Intake of digestible dry matter (DDM), CP (DCP), NDF (DNDF) and starch (Dstarch) were calculated by multiplying DMI by the digestibility coefficient of DM, CP, NDF and starch, respectively; FE was calculated as the ratio between average milk yield per cow and the average DMI and expressed as kg milk/kg DMI [3]. The IOFC in the milking-cow group was calculated by the following formula [4], using set values for the price of milk (0.4 EUR/kg) and the cost of TMR (0.24 EUR/kg DM):

(3) IOFC (EUR/cow/d)= daily average milk production (kg/cow/d)× milk price (€/Kg)− total feed cost (EUR/cow)/d)

The choice of setting the price per kg of milk and TMR was done on purpose to highlight the effect of conversion of feed into milk by animals and neglect the effects due to price volatility and market fluctuations. The SWR in milk yield was calculated to indicate management consistency along the year, especially considering the management of heat stress, and was attained by dividing for each farm the MY recorded during the 3 days preceding the summer visit by the same variables recorded during the 3 days preceding the winter visit of the same year [25]. The closer the SWR is to 1, the less effect the summer period, mainly characterised by the presence of heat stress, is having on milk production [25]. This calculation was possible because no farms had seasonal calving. The normal distribution of data was tested using the Shapiro–Wilk test (>0.90 = normally distributed). To verify the significance of the differences between the farms belonging to the three production classes, data on TMR composition, HI, average days in milk, SWR, diet digestibility, DDM, digestible nutrients, feed efficiency and IOFC were analysed through an analysis of variance (ANOVA) mixed model using the production class (three levels: H, M, and L) as a fixed effect and the farm nested in the production class as a random effect. Post-hoc pairwise comparisons were run between factor levels using Bonferroni correction. The assumptions of the linear model on the residuals were graphically tested. Simple linear regressions between DMI and MY and between DMI and DMD were run to assess the relationship between those variables, followed by a multiple linear regression to assess the relationships between the overall nutritional variables (DMI, nutrient content, digestibility of different nutrients, HI, DDM and digestible nutrients) and MY. To avoid redundancy, variables with a Pearson correlation coefficient ≥ 50% with the others were removed among nutritional variables. Feature selection was done using a forward step-wise algorithm, and the criteria for inclusion or exclusion was a *p*-value threshold of 0.15. To predict the achievable milk yield (AMY) of a farm which would be obtained by modifying only factors not related to feed (reducing factors), the regression model found was applied and the difference (Delta) between AMY and MY and its percentage value on MY were calculated for the different production classes. To verify what the AMY would be with no feed-limited factors (NFLMY), the regression model based on DDM and CP%, was run for each farm, replacing the original DDM value with that obtained by multiplying the farm’s DMI by the DMD value of 0.63. The latter is the average DMD value of the three (96 percentile) best producing farms. Then, the difference between NFLMY and MY and its percentage of the actual production was calculated. The same calculations were carried out for IOFC, by replacing MY with AMY and NFLMY, obtaining achievable (AIOFC) and not feed-limited IOFC (NFLIOFC). The same ANOVA model was run to verify the differences between production classes in AMY, NFLMY, AIOFC and NFLIOFC. Statistical analyses were performed using SAS release 9.4 (SAS Institute Inc., Cary, NC, USA, 2012).

## 3. Results

As reported in Table 3, the size, expressed as the number of cows in milk, and the average of herd’s days in milk did not differ among farms of the three production classes, whereas IOFC, taken as metric of economic performance, significantly increased from the L to the H production class.

The average chemical composition of the milking cows’ ration (Table 4) did not show significant differences among the classes, as well as the average digestibility of DM, CP, NDF and starch. The GMPL was not significantly different as well, whereas the homogeneity of the TMR along the feeding line, expressed as HI, tended to be significant and was higher in H farms.

As reported in Table 4, the variation of the ration between the different visits at the farm, expressed as CV %, among chemical and physical variables ranged from 2% for CP in L farms to 12% for ADL in L farms and did not show significant differences among production classes. An exception was given by HI which was significantly lower in H class compared with L and M classes.

With regard to performance (Table 5), milk yield, by definition, significantly increased from L to H classes, as well as FE, ranging on average from 1.32 to 1.53 kg milk/kg DMI. Farms belonging to H class on average showed significantly higher DMI, digestible dry matter (DDM), digestible crude protein (DCP) and CP intake (*p* = 0.084), compared with farms from other classes.

**Figure 1:**
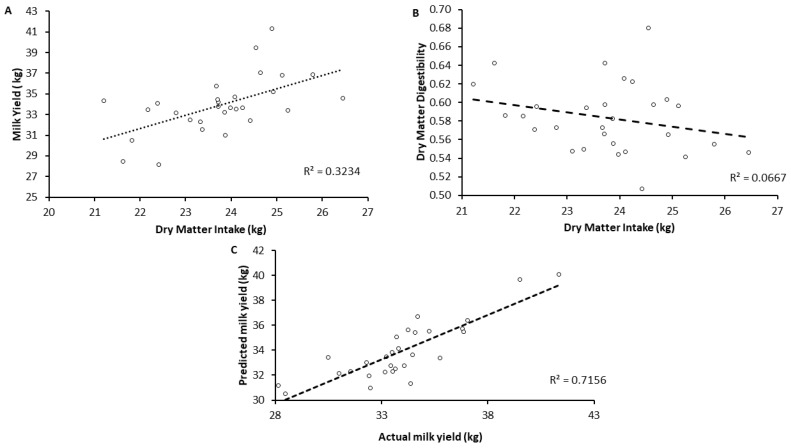
(**A**) Simple regression between dry matter intake and milk yield, (Mean SE = 5.45; R^2^ = 0.323; Adjusted R^2^ = 0.298); (**B**) Simple regression between dry matter intake and dry matter digestibility, (Mean SE = 13.48.45; R^2^= 0.067 Adjusted R^2^ = 0.032); (**C**) Multiple linear regression to estimate milk yield through digested dry matter (DDM) and crude protein content, (Mean SE = 2.38; R^2^ = 0.716; Adjusted R^2^ = 0.694).

Considering the conversion of DDM into milk (DDMconv), H and M farms showed higher milk yield than L farms. Regarding stepwise regression, among the nutritional variables, the explanatory variables selected were DDM and CP, which explained the milk yield with a R^2^ of 0.716 (*p* < 0.05), as reported in Table 6 and Figure 1.

**Figure 2:**
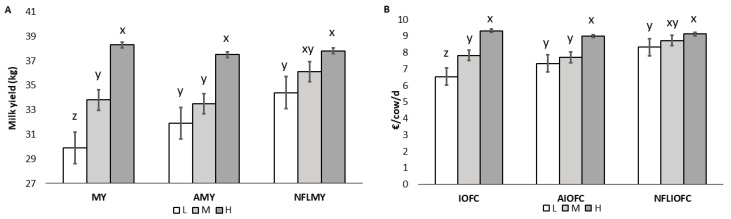
(**A**) Average milk yield (MY), achievable milk yield (AMY) and no feeding-limited milk yield (NFLMY) in low (L), medium (M) and high (H) production classes; (**B**) income over feed cost (IOFC), achievable income over feed cost (AIOFC) and no feeding-limited income over feed cost (NFLIOFC) in low (L), medium (M) and high (H) production classes. ^x, y, z^ Different letters between production classes stand for a significant difference (*p* < 0.05). Error bars represent the standard error for each production class.

The DDM alone explained 0.66 of variance. On the other hand, the simple regression between MY and DMI showed a lower coefficient of determination (R^2^ = 0.323; *p* = 0.001), whereas the regression between DMD and DMI was not significant (R^2^ = 0.067; *p* = 0.176). As reported in Figure 2, owing to the increase of milk production in NFLMY (+6.75%) by M farms and an increase of both AMY (1.99 kg, +6.75%) and NFLMY (4.5 kg, +15.0%) by L farms compared with MY, AMY showed the disappearance of statistical difference in the milk yield between L and M farms, and NFLMY led to the reduction of statistical difference both between L and M and between M and H farms. In the same way, the application of AMY and NFLMY would increase IOFC by 12 and 27% in L farms and NLFMY alone would instead increase IOFC by 11.5% in M farms. As seen for MY, even in the income over feed cost there is a progressive loss of significant difference between production classes from IOFC to NFLIOFC.

## 4. Discussion

With the purpose of estimating farms’ yield gap and finding indicators that, on a local scale, increase the farmers understanding of the main factors currently limiting milk yield, feed efficiency and IOFC, we compared data of 29 dairy farms belonging to one of the most important areas of Italian dairy farming and estimated the improvement they could achieve by modifying those factors. In the considered geographic area, farms are rather homogeneous regarding breed, housing and ration composition. Overall, the farms included in the study adequately represented the variability in size and milk yield typical of the farms raising Holstein cows in the most specialised provinces of Veneto (Vicenza, Padova and Verona) and Lombardy (Brescia, Cremona and Mantova) regions [16]. In these regions, farms with more than 100 cows represent more than 50% of total farms, and raise more than 75% of the cows. The higher estimated IOFC shown by the high producing farms was expected, since higher milk production usually leads to higher milk income [4], although IOFC is also affected by DMI and related costs. Such a difference in IOFC between groups is relevant because it would lead to an average increase of 420 (M vs. L), 486 (H vs. M) and 926 (H vs L) EUR/cow/lactation, considering the median value of 326 d per lactation reported by AIA [16]. However, it is important to remember that in this study, to highlight the effect of conversion of feed into milk by animals and neglect the effects due to price volatility and market fluctuations, as commonly found in the literature [5], the IOFC was calculated using pre-set realistic costs per kg of TMR and milk. This means that the difference in IOFC between L, M and H farms is due only to the difference in the conversion of feed into milk by milking cows. In addition, it must be considered that although IOFC affects farm profit, the latter is also determined by other factors such as reproductive efficiency, replacement rate and veterinary costs.

Average diet composition in all the classes follows the recent nutrient recommendations for early and mid-lactating Holstein cows [1] for crude protein (150–170 g/kg DM), NDF (280–320 g/kg DM) and starch (202–260 g/kg DM) and this is likely the reason why there are no significant differences among them. This also means that the importance of proper nutrition for lactating dairy cows has been correctly conveyed by nutritionists and understood by most farmers, even though, ideally, farmers might reach a better efficiency and IOFC by providing a customised TMR for different nutritional cluster groups instead of using a single TMR for all the cows in milk [26]. The TMR CP concentration at the lowest end of the recommended range is due to both the high price of the protein fraction and the effort to limit nitrogen excretion with faeces. The similar nutrient composition of rations based on maize silage and common ingredients of the ration led to a lack of significant difference in total tract digestibility of DM, CP, NDF and starch. Average digestibility values found in this study for DM are within the range reported in the literature, especially as regards high starch diets [12,27]. Whereas starch digestibility was slightly higher [12], CP and NDF digestibility were on average lower than those reported in the literature [12,27]. This difference is likely due to the frequent inclusion of cereal-based forages and straw in the diet. Even though diet formulation and preparation were similar among farm classes, a slightly higher HI value and a lower CV% of HI (Table 4) in H farms revealed a more homogenous chemical and physical TMR composition along the feed bunk and a higher TMR consistency over time. As reported by Serva et al. [10], the TMR homogeneity at the feed bunk and its consistency over time are of paramount importance in improving feed efficiency. In fact, increased variability in the TMR and cows’ habits of choosing the same position at the feed bunk [10] increase the difference in daily nutrients intake and alter the microbial community at the rumen level. This balance must be kept as consistent as possible over time [28] and although we are aware that a slight daily variation in the composition of the ration is inevitable, it has been found that the higher the variation in TMR nutrients, the higher the risk of compromising both production capacity and cow health [28].

This is in line with findings from another study, where a 0.5% and 5% increase in net energy and TMR long particle concentration changes were associated with a decrease of the daily milk production of 3.2 and 1.2 kg, respectively [29]. A similar result was found in young bulls, where a daily variation of starch and NDF of about 6%, compared with a variation of 3–4%, led to a 90 g reduction in daily gain and an increase in the food conversion ratio from 4.97 to 5.39 [9]. This effect is probably due to a greater efficiency in the use of nutrients by rumen microorganisms, which form balanced and efficient microbial communities in the presence of a constant supply of a balanced diet. Such microbial communities, if altered by an imbalance in feed resources, could in fact take up to 14 days to change and adapt to diet variation [30].

Analysing the data obtained from H and M farms, it can be noticed that the farms of both classes have the same conversion capacity of DDM into milk, equal to 2.47 kg milk/kg DDM. This value is very similar to what Potts et al. [27] reported in recent years for specialized dairy farms (2.45 milk kg/kg DDM). Increasing the DDM and in particular DCP, by increasing DMI and feed digestibility, should reduce the production gap between M and H farms. In this regard, the increase in DMI should not be obtained at the expense of feed digestibility, as it is sometimes reported in the literature [27]. The higher DMI of the H farms, excluding the chemical and physical composition of the ration which was not statistically different, can be attributed to the highest MY, which can also be considered a driver of DMI, and at least partially to feed management practices [14]. Frequent meals over a long period of time at the feed bunk, TMR delivery, feed push-up, return from milking and low feed bunk competition are in fact reported to increase DMI [14]. Prolonged milking time and the competition for stalls can also lead to a drop in feeding time due to a compensatory increase in lying time, and limit DMI, since cattle prioritize lying down over feeding [15]. Regarding the gap in FE between M and H farms, this could be due to multiple causes. At the individual level, Richardson and Herd [31] reported that the variation of feed efficiency in beef cattle could be attributed to differences in a range of factors including: digestibility (10%), feeding patterns and physical activity (12%), heat production (9%), body composition (5%), tissue turnover and metabolism (37%) and miscellaneous metabolic processes. Similar causes are also reported by Fisher et al. [32] for dairy cows. Another very important driver of the difference in FE is the dilution of nutritional needs for maintenance, which in high producing cows is higher, leading to a better FE [3]. In our study, differences in FE between herd classes could only partly be explained by digestibility, since the difference in digestibility between farm classes was not significant. On the other hand, management factors could at least contribute to part of the difference in efficiency between herds, including those related to TMR preparation and delivery, climate mitigation, management of transition cows, animal health and welfare [7,9,25]. In our case, the gap between M and H farms might be at least partially filled by improving TMR homogeneity and consistency over time, as already said above [30], but part of it is likely due to the dilution of the proportion of energy and nutrients diverted toward maintenance [3]. With regard to the differences between L and M farms, it is worth noticing that beside the lower FE, L farms have a lower conversion of DDM into milk which is independent from DMI, digestibility and consequently DDM. Possible explanations can be found in the different basal metabolic rate which affects the proportion of nutrients made available for milk production [3], and management differences which can lead to welfare and health issues. As reported by Elsasser [33] and Ingvartsen and Moyes [34], during stress events part of the available nutrients and energy can be diverted away from production and growth processes to metabolic pathways needed to return to homeostasis and involving endocrine hormones and immune system cytokine signals. In our study, an indirect measure of animal management differences between farm classes was given by the SWR in MY. Dairy cattle generally respond to heat stress through adaptation of physiological parameters, such as the respiration rate and decreasing milk production. The higher SWR in milk production in M and H farms could mean that the prevention and management of heat stress in summer, through the correct use of different heat abatement strategies, were very effective [25]; on the contrary, in L farms, which saw a 20% drop in production between winter and summer, the low value of SWR is possibly attributable to heat stress and other factors.

Overall, among the nutritional variables in this study, DDM, which is the combination of DMI and DM digestibility, and CP% in TMR explained together 71% of the variance in milk production. Although the positive relationship between milk production and DMI is well known [7], it has a lower coefficient of determination than the relationship between milk production and DDM. For this reason, the challenge for a farmer is to increase DMI without decreasing DM digestibility. For the protein concentration of the diet, its increase is often associated with increased milk yield and usually an increase in DDM, because CP is generally more digestible than fibre [7]. This is supported by the increase in DCP found in H farms and it is in line with the latest recommendations on nutrient requirements for early postpartum cows of 138 g/g of metabolizable protein/kg DM [1]. As regards reducing factors, in our study we measured only some indirect indicators of herd management such as the herd average days in milk and the SWR in milk yield. Measuring other parameters might give a more detailed insight into the differences in the DDM conversion into milk. The ratio between the number of cows and stalls, individual space at feed bunk, and cows’ time budget are good examples of how facilities and management practices can affect milk yield [14]. In addition, data on the overall health variables and the management of transition period, which is known to affect the incidence of post-partum disorders, might give an even more detailed insight into the causes of a low DDMconv [34]. In this regard, data on body condition score loss during the transition period, which indicate the level of body fat mobilization, would also help in explaining differences in FE and other farm performance indicators such as reproductive parameters and replacement percentage [35].

As reported in Figure 2, running the multiple regression model giving the prediction of milk yield and AMY, on the basis of DDM and CP% of the ration, did not show any significant differences compared with actual milk yield for farms belonging to M or H classes, but increased milk yield by 6.75% in L farms. This finding means that low production farms compared with the M class have a further yield gap due to low conversion of DDM into milk, that is likely attributable to management or health factors. However, since this gap is conspicuous, although all the farms raised Italian Holstein cows and this breed has recently shown a loss in genetic diversity [36], it cannot be ruled out that genetics has at least partly contributed to the milk production difference. On the other hand, calculating NFLMY by replacing the real DDM with the one calculated using the DMD of the best 96 percentile farms led to an important reduction of milk gap among farm classes, underlying the importance of feed-limited factors, well summarized by DDM. The same trend was reported for IOFC, AIOFC and NFLIOFC, since their calculation is based on MY, AMY and NFLMY, respectively. The yield gap between potential production, feed limited production and actual production for dairy cows was already described by Van Der Linden et al. [7] who at the individual cow level confirmed the importance of protein deficiency (39.4%), feed intake capacity (23.5%) and heat stress (1.2%). In their study, however, the authors focused only on healthy individuals and did not consider the effects of reducing factors such as health issues, stressors other than heat stress or inter-farm differences in management procedures. Those reducing factors instead appear to have a different weight in the explanation of yield gap in different farms in our research. A detailed yield gap analysis at the herd level might in fact be helpful to define and explore options to maximize the performance, improve resource use efficiency and decrease the gap between actual and potential production of a farm [6,7]. This study, despite some limitations, gives some insights on indicators and suggestions which might help farmers in decision making and be a basis to build or improve complex mechanistic models.

### Limitations and Recommendations for Future Research

Although the present research shows the potential usefulness of important indicators such as HI variation, DDM, or the conversion capacity of DDM into milk in the detection of the herd-level production limiting factors, some limitations exist. The research focused on a specific production area where farms were characterized by a single group of lactating cows and raised cows permanently indoors. Despite the cows all being Italian Holsteins, the differences in cow genetics between farms were not studied. Milk composition was not available for all farms and for this reason milk yield was expressed as kg of milk and not as fat- and protein-corrected milk. Indices related to animal management were limited to average days in milk and SWR. Furthermore, the yield gap analysis regarding NFLMY was based on the DMD of the three best producing H farms. This means that the suggested limiting factors mainly refer to L and M farms, but only partially to H farms. Future research should consider farms with more than one lactation group and belonging to different dairy production systems and use fat-corrected or energy corrected milk. Details on facilities, genetics, animal management procedures, cows’ time budget and health status must be studied to be able to better investigate the causes of low DDM conversion into milk.

## 5. Conclusions

In conclusion, this yield gap analysis in a sample of dairy farms belonging to a specialized dairy production area of Northern Italy gave an interesting insight into the main constraints of farms belonging to different production classes. Contrary to our expectations, there were no significant differences in ration composition between farm classes, whereas the main nutritional factors discriminating between the high production farms and the others were dry matter intake, digestible dry matter, digestible crude protein and TMR consistency over time. Low and medium production farms had similar dry matter intake and digestible dry matter, but L farms showed a lower conversion of digestible dry matter into milk, likely due to reducing factors such as health issues, distress and management procedures. Of the factors examined in the study, digestible dry matter and crude protein content explained together 71% of the variance in milk production. Both factors were used to estimate the achievable production, attainable without reducing factors constraints, and the no feed-limited milk yield through the optimization of the digestible dry matter. Despite some limitations, this study suggests some indicators and a practical approach which might help farmers estimate their yield gap and understand specific milk yield, feed efficiency and IOFC limitations. Further research with a deeper insight on reducing factors and involving a wider range of dairy farming systems is warranted.

## Figures and Tables

**Table 1:** Average, standard deviation (SD), minimum (Min) and maximum (Max) values of the main characteristics and performance of the recruited farms.

	Average	SD	Min	Max
Milking cows (n)	289	207	49.0	483
Milk yield (kg/cow/day)	33.9	2.79	28.1	41.3
DMI (kg)	23.8	1.24	21.2	26.4
FE (milk kg/kg DMI)	1.43	0.097	1.25	1.66
IOFC (EUR/cow/day)	7.86	0.976	5.88	10.6

DMI = dry matter intake; FE = Feed efficiency; IOFC = income over feed cost; Farms number = 29. Methods used to calculate the above parameters are reported below (Section 2.3 and Section 2.5).

**Table 2:** Specifications of the PoliSPEC^NIR^ calibration curves developed and used for the calculation of homogeneity index (HI) of the total mixed ration (TMR) and specifications of the FOSS DS-2500 calibration curves used to measure the chemical composition of faeces and the chemical and physical composition of TMR.

Constituents	N of Samples	SECV	1-VR
PoliSPEC^NIR^			
DM (g/kg)	1392	19.3	0.97
CP (g/kg DM)	1394	8.10	0.79
NDF (g/kg DM)	1275	18.8	0.86
Starch (g/kg DM)	1384	15.7	0.78
^1^ S4 (%)	316	6.25	0.56
^1^ S5 (%)	312	3.15	0.56
Bottom pan (%)	311	4.40	0.69
GMPL (mm)	309	2.46	0.71
FOSS DS-2500			
TMR			
DM (g/kg)	345	12.6	0.97
Ash (g/kg DM)	341	3.80	0.82
CP (g/kg DM)	341	5.60	0.81
NDF (g/kg DM)	343	13.5	0.89
ADL (g/kg DM)	293	3.60	0.54
Starch (g/kg DM)	342	14.4	0.71
Faeces			
DM (g/kg)	135	5.60	0.75
Ash (g/kg DM)	135	7.70	0.86
CP (g/kg DM)	135	5.30	0.91
NDF (g/kg DM)	343	23.7	0.82
ADL (g/kg DM)	126	5.00	0.84
Starch (g/kg DM)	132	9.20	0.84

SECV = standard error of cross-validation; 1-VR = coefficient of determination in cross-validation; GMPL = geometric mean particle length. ^1^ Sieves 4 (3.8 mm) and 5 (1.8 mm).

**Table 3:** Size, average days in milk and income over feed cost (IOFC) of the farms belonging to the low (L), medium (M) and high (H) production classes.

	L	M	H	SEM	*p*-Value ^1^
Cows in milk (N°)	263	299	281	56.8	0.284
Herd average days in milk (d)	169	173	174	5.03	0.830
IOFC (EUR/cow/day)	6.54 ^z^	7.83 ^y^	9.32 ^x^	0.198	<0.001

^1^ Different letters (x, y, z) between production classes indicate a significant difference (*p* < 0.05).

**Table 4:** Total mixed ration (TMR) average chemical and physical characteristics, total tract digestibility and coefficient of variation for farms belonging to low (L), medium (M) and high (H) production classes.

	L	M	H	SEM	*p*-Value ^1^
Composition (g/kg DM)					
DM	508	505	480	12.7	0.152
CP	148	149	149	1.59	0.833
NDF	341	332	324	5.50	0.215
ADL	33.3	31.0	30.8	1.43	0.462
Starch	255	258	262	5.96	0.789
Digestibility					
DM	0.595	0.574	0.607	0.013	0.171
CP	0.557	0.561	0.594	0.018	0.350
NDF	0.320	0.315	0.351	0.021	0.449
Starch	0.980	0.975	0.981	0.003	0.265
Physical characteristics and homogeneity of the ration					
GMPL (mm)	6.14	6.04	6.45	0.179	0.244
HI (pure number)	72.7	73.1	78.2	1.73	0.090
CV (%) of rations					
DM	4.18	4.18	4.69	0.854	0.903
CP	2.04	3.08	4.21	0.598	0.121
NDF	4.22	4.40	4.88	0.882	0.896
ADL	12.0	11.1	11.0	2.03	0.946
Starch	6.24	4.95	4.37	1.22	0.643
CV (%) of physical characteristics and homogeneity of the ration					
GMPL (mm)	9.89	11.28	11.74	1.94	0.830
HI (pure number)	15.4 ^x^	14.8 ^x^	5.60 ^y^	2.64	0.040

GMPL = geometric mean particle length; HI = homogeneity index. ^1^ Different letters (x, y) between production classes indicate a significant difference (*p* < 0.05).

**Table 5:** Average performance, expressed as production, feed intake, efficiency and summer–winter ratio (SWR) in farms belonging to low (L), medium (M) and high (H) production classes.

Performance	L	M	H	SEM	*p*-Value ^1^
Production (kg)					
Milk yield	29.9 ^z^	33.8 ^y^	38.3 ^x^	0.472	<0.001
Feed intake (kg)					
DM	22.6 ^y^	23.7 ^y^	25.0 ^x^	0.395	0.006
DDM	13.0 ^y^	13.7 ^y^	15.5 ^x^	0.292	<0.001
CP intake	3.38	3.52	3.68	0.070	0.084
DCP	1.92	1.97	2.20	0.061	0.019
NDF	7.81	7.94	8.04	0.191	0.762
DNDF	2.54	2.63	2.89	0.246	0.670
Starch	5.85	6.14	6.45	0.173	0.142
Dstarch	5.73	5.97	6.33	0.175	0.142
Efficiency					
FE (kg milk/kg DMI)	1.32 ^z^	1.43 ^y^	1.53 ^x^	0.029	0.001
DDMconv (kg milk/kg DDM)	2.29 ^y^	2.47 ^x^	2.47 ^x^	0.04	0.009
Milk yield SWR (pure number)	0.88	0.92	1.04	0.037	0.041

DMI = dry matter intake; DDM = digested dry matter; DCP = digested crude protein; DNDF = digested neutral detergent fibre; Dstarch = digested starch; GFE = gross feed efficiency; DDMconv = conversion of digested dry matter into milk; SWR = summer–winter ratio. ^1^ Different letters (x, y, z) between production classes indicate a significant difference (*p* < 0.05).

**Table 6:** Metrics of the multiple regression analysis with the milk yield as dependent variable and digestible dry matter (DDM, kg) and crude protein (%) as predictors.

Variables	Coefficients	R² Partial	R² Total	*p*-Value
Intercept	−19.0914			
DDM	2.13188	0.6619	0.6619	<0.001
CP	1.57349	0.0537	0.7156	0.036

DDM = digestible dry matter.

## Data Availability

The data presented in this study are available on request from the corresponding author. The data are not publicly available since they are still under analysis for further publications.
